# Properties of the major Zn^2+^-binding site of human alpha-fetoprotein, a potential foetal plasma zinc carrier†

**DOI:** 10.1039/d4cc06611a

**Published:** 2024-12-27

**Authors:** Jin Lu, Stephen J. Hierons, Swati Arya, Remi Fritzen, Sirilata Polepalli, Siavash Khazaipoul, Alan J. Stewart, Claudia A. Blindauer

**Affiliations:** a Department of Chemistry, University of Warwick Coventry CV4 7AL UK c.blindauer@warwick.ac.uk; b School of Medicine, University of St. Andrews St. Andrews KY16 9TF UK ajs21@st-andrews.ac.uk

## Abstract

The foetal plasma protein α-fetoprotein (AFP) harbours a high-affinity zinc binding site that is likely involved in transport and delivery of essential zinc during foetal development. Based on a recent electron microscopy structure of AFP and aided by biophysical studies on an AFP-derived peptide, we present a refined 5-coordinate model for this site.

## Abstract 2

The foetal plasma protein alpha-fetoprotein harbours a strong Zn^2+^ binding site (*K*_D_ = 1.9 × 10^−8^ M), supporting the hypothesis of a role in foetal zinc homeostasis.

## Graphical abstract

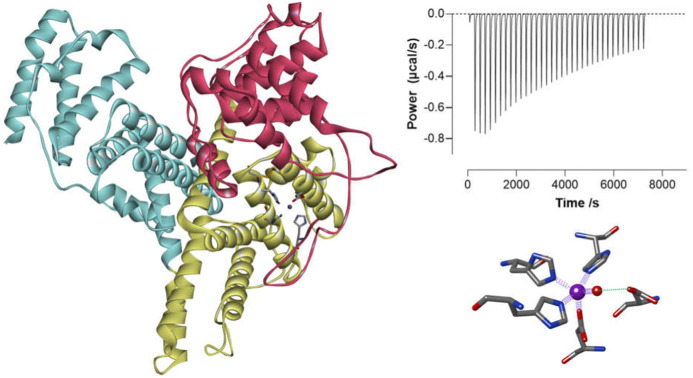

Human α-fetoprotein (AFP) is produced by the foetal liver and is the most abundant protein in foetal plasma (*ca.* 1–10 mg mL^−1^) during certain stages of development.^1,2^ Although AFP has long been used as a diagnostic tool in prenatal care and liver cancer detection, the physiological roles of this protein are only partially understood.^3–5^

Human AFP shares 39.4% sequence identity with human serum albumin (HSA), with each composed of three homologous domains (I–III).^3,4^ The plasma concentration of AFP decreases sharply after birth to trace amounts, with HSA replacing it as the major plasma protein.^6^ Both AFP and HSA bind many of the same ligands, which include fatty acids,^7,8^ bilirubin,^9^ and divalent metal ions,^10,11^ suggesting overlapping functions. The affinities of AFP for these cargoes are typically one to two orders of magnitude higher than those of HSA; this is also the case for the binding of Zn^2+^.^11,12^

An adequate Zn^2+^ supply is vital for normal foetal development in mammals, as demonstrated by the teratogenicity of maternal zinc deficiency.^13,14^ However, the mechanisms by which Zn^2+^ is delivered to the foetus and taken up by foetal tissues are poorly understood, and no comprehensive model has been proposed. Several studies have reported an association between Zn^2+^ and AFP in amniotic fluid.^15,16^ This supports the hypothesis that AFP – like its relative HSA – binds Zn^2+^ under physiological conditions. The significance of HSA as the most critical player in regulating the availability of zinc in blood plasma is becoming increasingly clear.^17–19^ We hypothesise that AFP plays a similar role in foetal plasma. The zinc-binding ability of AFP has been confirmed *in vitro*,^11,20^ with the more recent study reporting a single high-affinity Zn^2+^ binding site (*K*_D_ < 10^−8^ M) and at least four lower-affinity sites (*K*_D_ < 10^−5^ M). Given that the pH-independent *K*_D_ value for the high-affinity Zn^2+^ site on HSA is reported to be *ca.* 10^7^,^21^ AFP is a plausible foetal plasma Zn^2+^ carrier and may participate in Zn^2+^ homeostasis during foetal development.

In 2023 the first 3D-structure of human AFP was elucidated using cryo-EM.^22^ More recently a second AFP structure was reported with Zn^2+^ and fatty acids bound, identifying the location and structure of a single Zn^2+^ binding site on AFP.^23^ The Zn^2+^ site appears to be 4-coordinate with ligands provided by the sidechains of His4, His246, His250, and Asp262 residues (the amino acid numbering used relates to the mature protein (Fig. 1). Three of the four residues (His246, His250, and Asp262) reside within a helix–loop–helix region in domain II of the protein.

**Fig. 1:**
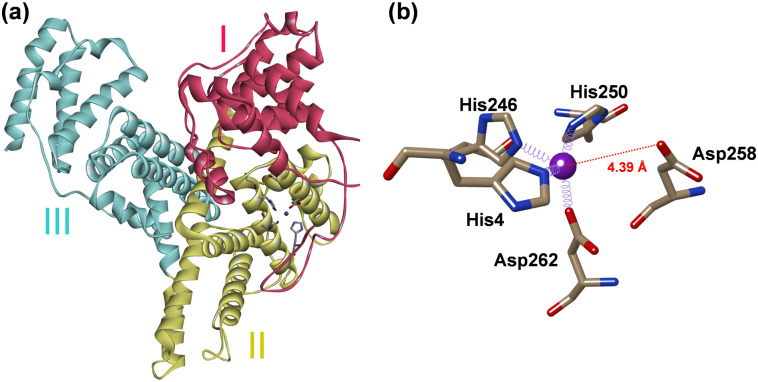
(a) Cryo-EM structure of human AFP coloured by domain (I–III drawn from PDB: 8X1N).^23^ (b) Close-up of the Zn^2+^-binding site. The site is composed of N–ligands from His4 (domain I), His246 and His250 and an O–ligand from Asp262 (domain II).

A closer inspection of the Zn^2+^ binding site modelled in the cryo-EM structure (Fig. 1(b) and Fig. S1, ESI†) indicates that its coordination sphere is incomplete or distorted (the authors suggested that this may be due to the site being occupied by multiple different metal ions in their sample^23^). In particular, the angles between the coordinating atoms more closely resemble a trigonal bipyramidal geometry, with His246 at one of the apices, and the other apex left empty. In addition, the angles between the aromatic ring planes of His4 and His250 and their bonds to the Zn^2+^ ion also deviate significantly from the expected 180°, and all four Zn–ligand bond lengths are much longer than expected. We attempted to re-model the site with a rotated Asp258 sidechain, and whilst this brought one of the O atoms within binding distance, comparison with the electron microscopy dataset (EMD) map did not support this model (not shown).

To explore the Zn^2+^-binding properties of AFP further, we have studied the Zn^2+^ affinity of recombinant AFP in a system previously used for HSA to allow direct comparison. In addition, we have also characterised a synthetic peptide corresponding to the helix–loop–helix region. It was necessary to treat the expressed protein with EDTA and extensively dialyse it to remove metal ions (Zn^2+^, Cu^2+^ and Fe^2+/3+^), presumably picked up during expression and/or purification. Isothermal titration calorimetry (ITC) was employed to examine Zn^2+^-binding to recombinant human AFP (Fig. 2). The binding of Zn^2+^ was exothermic (Table S1 and Fig. S2, ESI†) and the resultant data could be fitted using a “two sets of sites model” with the stoichiometry of site 2 fixed to 1 (Fig. 2). Models involving a single site or with varying site 2 stoichiometry gave unsatisfactory fits (Table S1, ESI†).

**Fig. 2:**
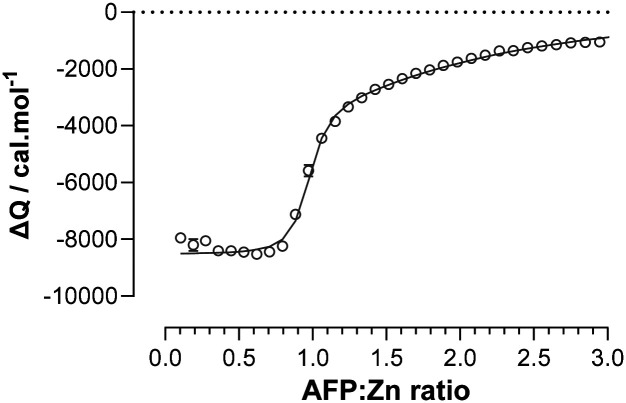
ITC data for Zn^2+^ binding to recombinant human AFP. Using a MicroCal VP-ITC instrument, 32 injections of 5 μL of 300 μM ZnCl_2_ were delivered to samples of 20 μM AFP in buffer containing 50 mM Tris, 140 mM NaCl, pH 7.4 (298 K). The solid line represents the best fit to experimental points using a model involving two sets of sites.

The data gave an apparent binding constant of log *K*_appITC_(Zn-AFP) = 7.4 ± 0.1 with a stoichiometry of 0.93 ± 0.01 for site 1 and a log *K*_appITC_(Zn-AFP) = 4.8 ± 0.1 for site 2. Correction for competition with 50 mM Tris gave an apparent binding constant of log *K*_app_(Zn-AFP) = 7.8 ± 0.1, valid at pH 7.4, which translates to *K*_D_ = 1.9 × 10^−8^ M. This is two orders of magnitude lower than the analogous apparent *K*_D_ for HSA (2.9 × 10^−6^ M, also corrected for competition with Tris).^12^

The helix–loop–helix region that harbours three of the proposed Zn^2+^-binding ligands has additional residues with Zn^2+^-binding ability, including His248, Glu249, Asp255 and Asp258. In the cryo-EM structure, a carboxylate oxygen of Asp258 is at a distance of only 4.4 Å to the Zn^2+^ ion (Fig. 1(b)). To explore whether alternative binding modes might be possible in this region, we designed a synthetic peptide corresponding to residues 245–263 of mature AFP. The peptide was synthesised commercially (95% purity; Genscript, Piscataway, NJ, USA). The N-terminus was acetylated, and the C-terminus was amidated to prevent the amine and the carboxylate from participating in metal binding. Cys251 and Cys259 form a disulfide bond in full-length AFP, whilst Cys252 forms a disulfide bond with Cys206. Therefore, Cys252 was substituted with a serine, to avoid formation of erroneous disulfide bonds and the remaining third Cys acting as a metal ligand (Fig. 3(a)). The identity and oxidation state of the AFP-peptide was confirmed using electrospray ionisation mass spectrometry (ESI-MS). The mass data (Table S2 and Fig. S3, ESI†) are consistent with those expected for oxidised peptide (*i.e.* disulfide bond formed), with the mono-isotopic neutral mass observed at 2128.99 Da, in excellent agreement with the theoretical mass (2128.92 Da). The close match between experimental and theoretical isotopic distributions (Fig. S3, ESI†) suggests that no reduced species were present.

**Fig. 3:**
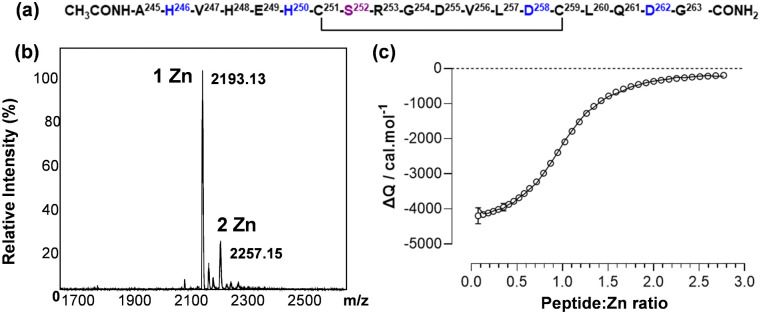
(a) Amino acid sequence of the synthetic peptide designed to mimic the helix–loop–helix motif that forms the major part of the Zn^2+^ site of AFP. The superscript numbers give the positions of the corresponding amino residues in mature AFP. Residues proposed to be involved in Zn^2+^-binding are coloured in blue and the Ser substitution (in place of Cys252) is in purple. (b) Deconvoluted ESI-MS spectrum (MicrOTOF, Bruker Daltonics) of the Zn^2+^-loaded AFP-peptide (10 mM ammonium acetate, pH 7.4, 10% methanol). The signals correspond to two distinct forms of the metalated-AFP-peptide, with one or two Zn^2+^ ions bound. (c) ITC data for the addition of ZnCl_2_ (333 μM) to AFP-peptide (25 μM) in 50 mM Tris buffer with 50 mM NaCl, pH 7.2. The solid line represents the best fit to experimental points using the model involving two sets of sites.

The Zn^2+^-binding ability and stoichiometry were assessed using native ESI-MS and inductively-coupled plasma optical emission spectroscopy (ICP-OES). ICP-OES analysis gave a Zn-to-peptide ratio of 1.2 ± 0.1. Native ESI-MS revealed the presence of two Zn–peptide species. The most abundant isotopic peak for the predominant species in the deconvoluted mass spectrum of the Zn^2+^–peptide sample had a neutral mass of 2193.10 Da (Fig. 3(b)), which matches the theoretical most abundant isotopic peak for the Zn_1_–peptide metalloform (2192.83 Da). A second species, Zn_2_–peptide (most abundant neutral mass 2257.07 Da; theoretical mass 2256.76 Da) was also observed in the mass spectrum as a minor constituent. This agrees with the ICP-OES analysis; thus, both methods indicated that a 1 : 1 species is formed predominantly, but also that more than one equivalent of Zn^2+^ can bind to the AFP-peptide with appreciable affinity.

ITC was used to determine the affinity for Zn^2+^ binding to the AFP-peptide (Fig. 3(c)). As observed with recombinant AFP, the binding of Zn^2+^ to the AFP-peptide was exothermic (Fig. S4, ESI†). Using a “one set of sites” model to fit the data gave an apparent binding constant of log *K*_appITC_(Zn–peptide) = 5.7 ± 0.1 with a stoichiometry of 1.05 ± 0.01. Since ICP-OES and ESI-MS data indicated the possible existence of a second binding site, models involving two (sets of) binding sites were also explored. Using these models, apparent binding constants of log *K*_appITC_(Zn–peptide) = 6.1 ± 0.1 for site 1, and 4.6 ± 0.1 for site 2 were obtained. This model improved the curve fitting (goodness-of-fit improved from 75% for the single-site models to 91%), consistent with the existence of a second, much weaker binding site beside a strong primary site. It is however also important to note that the inclusion of a second site had only a relatively small effect on log *K*_appITC_ for the first site. Correction for the competition for Tris gave log *K*_app_ = 6.0 or 6.4, respectively, translating to an apparent *K*_D_ = 1.1 × 10^−6^ M or 3.9 × 10^−7^ M. Hence, Zn^2+^ binding to the peptide was 20 to 60 times less strong than to the full-length AFP protein, but in a similar range as that to HSA. Given that HSA harbours a site with only three protein-derived ligands (two His and one Asp residue),^24^ binding to the peptide is also likely to occur through (at least) three ligands, also considering that the fourth proposed ligand, His4, is absent.

The interaction between Zn^2+^ and the AFP-peptide was further studied by 1D and 2D ^1^H NMR spectroscopy. A series of ^1^H NMR spectra were acquired for the apo-AFP-peptide at pH 7.1 and various temperatures between 278 K to 310 K (Fig. S5, ESI†). At 278 K, there was a significant increase in the dispersion of the backbone amide NH resonances, with sharper and more resolved peaks being discernible, presumably because of slowed-down structural dynamics. All subsequent NMR experiments were carried out at 278 K. 2D TOCSY and NOESY data were acquired for the apo peptide as well as for a sample after addition of 1 mol. eq. of Zn^2+^, at pH 7.1. An overlay of the fingerprint region of the TOCSY spectra is shown in Fig. 4.

**Fig. 4:**
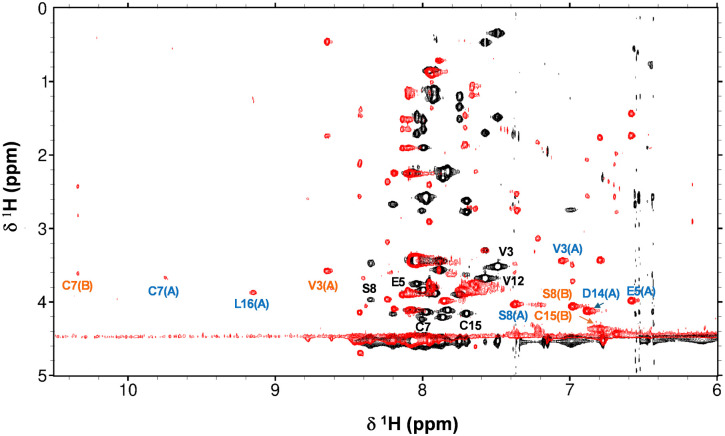
Overlay of fingerprint region of 2D TOCSY NMR spectra of the AFP peptide in absence (black) and presence (red) of 1 mol. eq. Zn^2+^ (700 MHz, 50 mM [D_11_]Tris–Cl, 50 mM NaCl, pH 7.1 and 10% D_2_O, 278 K). Selected residues are labelled in black (apo form), blue (species A) and orange (species B).

Strikingly, upon Zn^2+^ addition, the dispersion of NH resonances increased greatly, with new NH signals appearing in both the low-field (*e.g.* 3 signals at >9 ppm) and the high-field (several signals below 7.5 ppm) region. This indicates that the binding of Zn^2+^ drives significant structural changes in the peptide, with most of the NH and H(α) protons having moved into a different electronic environment.

Sequential assignment for both apo-AFP-peptide and the peptide in presence of 1 mol. eq. Zn^2+^ were established through the 2D TOCSY and NOESY spectra (Tables S4 to S6, ESI†). The chemical shifts for H(α) protons in the apo AFP-peptide were compared with random coil shifts (Fig. S6(a), ESI†). All H(α) protons possess chemical shifts that are substantially below random-coil shifts; this is consistent with α-helical character^25^ and in line with expectations based on the 3D structure of the full-length AFP protein.^23^ Sequential assignment of the spectra recorded in the presence of Zn^2+^ revealed the existence of two distinct species (designated A and B). Despite the substantial changes in chemical shifts compared to the apo form (Fig. S7, ESI†), α-helical character is largely maintained in both species (Fig. S6(b) and (c), ESI†). Since the volumes of the cross-peaks for these two species do not differ significantly, it can be assumed that they exist at broadly similar concentrations. As only 1 mol. eq. of Zn^2+^ had been added, and no resonances for the apo-form remained, it follows that both species have just one Zn^2+^ ion bound. Whilst in theory, the two species could just be conformers, it appears more likely that the peptide harbours at least two Zn^2+^ binding sites with similar affinity, but (with consideration of the ITC results which indicated only one strong binding site) that they are mutually exclusive.

To explore possible binding site compositions, AlphaFold modelling was used to generate starting models for 1 : 1 complexes of the AFP peptide with Zn^2+^. Analysis of these models suggested that in the peptide, a site comprising His248, His250 and Asp262 may be most favourable, whilst another model showed Zn^2+^ in proximity to His250, Asp258 and Asp262. Three of these starting models were selected for further refinement. To generate final models, tetrahedral coordination was completed by a water molecule in all cases, and geometries were optimised by energy minimisation (UCSF Chimera v. 1.11, Amber forcefield). The models (Fig. S8(a)–(c), ESI†) suggest that the zinc-bound water molecule could form a hydrogen bond to the carboxylate group of either Asp255 or Asp258. In addition, we used the cryo-EM structure to create two starting models harbouring a site composed of His246, His250 and Asp262 and either one or two waters. Whilst the model with one water failed to yield reasonable geometries, the 5-coordinate model was viable Fig. S8(d), ESI†). Attempts to generate 4-coordinate models involving His246, His250, Asp255 and Asp258 simultaneously did not generate satisfactory geometries. Together, ITC, NMR and modelling results point towards the existence of two alternative binding sites with similar affinities, involving three peptide-derived ligands.

Finally, given the apparently incomplete coordination sphere in the cryo-EM model, we generated a model of full-length AFP with the original four ligands plus a water molecule in the empty site (Fig. 5). Refinement of this model did not require any discernible changes of the protein backbone, and only minor adjustments of the sidechain conformations, mainly to improve distances and angles (Table S7, ESI†). The resultant model has a distorted trigonal-bipyramidal geometry and features an H-bond from the zinc-bound water to Asp258.

**Fig. 5:**
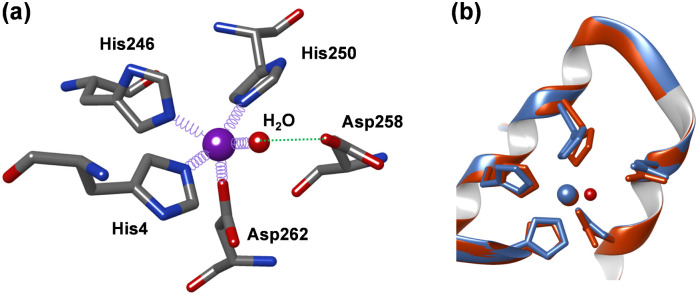
Optimised geometry for the Zn^2+^-binding site in AFP. (a) The model was optimised using pdb 8X1N as starting point, and after filling the empty coordination site with a water molecule. Angles and distances are summarised in Table S5 (ESI†). (b) Overlay of original structure (blue) and optimised model (red). The model and images were generated in Chimera v. 1.11.^26^

The work presented supports the recent identification of a novel Zn^2+^ binding region on human AFP that may be important for the transport of zinc in the foetal circulation. In comparison to the major Zn^2+^-binding site in adult plasma, the site in AFP features an additional histidine residue, which is consistent with its higher binding affinity (*K*_D_ = 1.9 × 10^−8^ M *vs.* 2.9 × 10^−6^ M under identical conditions). The AFP concentration in foetal plasma (15–150 μM) is 4 to 40-times lower than that of HSA in adult plasma (*ca.* 650 μM). Using a typical total [Zn] of 14 μM^27^ and [AFP] = 15 μM, [Zn]_free_ = 196 nM can be calculated (based on log *K* = 7.77). This reduces to less than 2 nM when 150 μM AFP are present. Although it is clear that other plasma proteins will further reduce [Zn]_free_, these simple estimates have several implications for zinc distribution to and within the foetus: transport through membranes depends on [Zn^2+^]_free_;^19^ therefore, at low AFP concentrations, it may be expected that transfer from foetal plasma into cells is enhanced. This will be advantageous for rapidly dividing cells and developing tissues within the foetus, which have a high requirement for zinc. At high [AFP], [Zn^2+^]_free_ in the foetal circulation will be lower than that in adult plasma (At 650 μM HSA and 14 μM total Zn, [Zn]_free_ = 63 nM). It is possible that lower [Zn^2+^]_free_ on the foetal side of the placental syncytiotrophoblast promotes mother-to-foetus zinc transfer – somewhat analogously to the higher affinity of foetal haemoglobin toward oxygen.

It is also worth noting that the Zn^2+^-binding ability of albumin is modulated by fatty acid (FA) binding.^18,28,29^ The AFP structure,^23^ which has Zn^2+^ and FAs bound simultaneously, provides strong evidence that Zn^2+^-binding to the primary site on AFP is unlikely to be affected by FA binding. While FA concentrations in foetal plasma are around 70–80% lower than in adult plasma,^30^ owing to its lower concentration the average FA loading of AFP likely is significantly higher than that of HSA. The physiological reason behind the allosteric interaction between zinc and FA binding on albumin has remained enigmatic, but it would appear that this crosstalk is not part of foetal physiology, and that foetal plasma zinc speciation and trafficking is independent of FA status.

A deeper understanding of AFP and how it affects zinc speciation and trafficking may aid our understanding of how aberrant AFP levels or alteration in plasma Zn^2+^ concentrations may contribute to developmental disorders.

This work was financially supported by the Leverhulme Trust (RPG-2017-214) and BBSRC (BB/J006467/1 and BB/V014684/1).

## Data availability

Data supporting this article are included in the ESI.†

## Conflicts of interest

There are no conflicts to declare.

## Supplementary Material

CC-061-D4CC06611A-s001

## References

[cit1] Bergstrand C. G., Czar B. (1956). Scand. J. Clin. Lab. Invest..

[cit2] Ruoslahti E., Seppälä M. (1972). Nature.

[cit3] Deutsch H. F. (1991). Adv. Cancer Res..

[cit4] Mizejewski G. L. (2004). Exp. Biol. Med..

[cit5] Yeo Y. H. (2024). et al.. Hepatol. Commun..

[cit6] Schieving J. H. (2014). et al.. Eur. J. Paediatr. Neurol..

[cit7] Parmelee D. C. (1978). et al.. J. Biol. Chem..

[cit8] Hsia J. C. (1980). et al.. J. Biol. Chem..

[cit9] Ruoslahti E. (1979). et al.. Biochim. Biophys. Acta.

[cit10] Aoyagi Y. (1978). et al.. Cancer Res..

[cit11] Permyakov S. E. (2002). et al.. Biochim. Biophys. Acta.

[cit12] Kassaar O. (2015). et al.. J. Thromb. Haemostasis.

[cit13] Uriu-Adams J. Y., Keen C. L. (2010). Birth Defects Res., Part B.

[cit14] Brion L. P. (2021). et al.. Pediatr. Res..

[cit15] Parkinson C. E. (1981). et al.. J. Obstet. Gynaecol..

[cit16] Laitinen R. (1985). et al.. Am. J. Obstet. Gynecol..

[cit17] Coverdale J. P. C. (2019). et al.. Metallomics.

[cit18] Sobczak A. I. S. (2021). et al.. Chem. Sci..

[cit19] Coverdale J. P. C. (2022). et al.. Chem. Commun..

[cit20] Wu J. T. (1987). et al.. Clin. Physiol. Biochem..

[cit21] Bal W. (2013). et al.. Biochim. Biophys. Acta, Gen. Subj..

[cit22] Zheng L. (2023). et al.. Nat. Methods.

[cit23] Liu K. (2024). et al.. Commun. Biol..

[cit24] Handing K. B. (2016). et al.. Chem. Sci..

[cit25] Wishart D. S. (2011). Prog. Nucl. Magn. Reson. Spectrosc..

[cit26] Pettersen E. F. (2004). et al.. J. Comput. Chem..

[cit27] Marsál K., Furgyik S. (1987). Acta Obstet. Gynecol. Scand..

[cit28] Coverdale J. P. C. (2019). et al.. Biochim. Biophys. Acta, Mol. Cell Biol. Lipids.

[cit29] Wu D. (2024). et al.. J. Lipid Res..

[cit30] Yu H. T. (2022). et al.. Front. Nutr..

