# Supplementary material for: Properties of the major Zn2+-binding site of human alpha-fetoprotein, a potential foetal plasma zinc carrier
Source: Chem Commun (Camb). 2024 Dec 27;61(10):2099–102. doi: 10.1039/d4cc06611a (PMC11726181; doi:10.1039/d4cc06611a)
Supplement: CC-061-D4CC06611A-s001 [file CC-061-D4CC06611A-s001.pdf]

This journal is © The Royal Society of Chemistry 2024

Properties of the major Zn^{2+}-binding site of human alpha-fetoprotein, a potential foetal plasma zinc carrier

Jin Lu, Stephen J. Hierons, Swati Arya, Remi Fritzen, Sirilata Polepalli, Siavash Khazaipoul, Alan J. Stewart, and Claudia. A. Blindauer

Supplementary Materials

Materials and Methods

Table S1. ITC data for full-length AFP.

Table S2. Observed and theoretical masses for apo and zinc-bound AFP peptide.

Table S3. ITC data for AFP peptide.

Table S4. ^{1}H NMR chemical shifts for apo peptide.

Table S5. ^{1}H NMR chemical shifts for Zn-bound species A.

Table S6. ^{1}H NMR chemical shifts for Zn-bound species B.

Table S7. Angles and distances for zinc-binding site in full-length AFP after force-field-driven optimisation of geometry through energy minimisation.

Figure S1. The geometry (angles and distances) of the modelled Zn^{2+} site in pdb 8X1N.

Figure S2. Thermogram for titration of full-length AFP with Zn^{2+}.

Figure S4. Thermogram for titration of AFP peptide with Zn^{2+}

Figure S3. ESI-MS analysis of AFP peptide in its apo form and after incubation with Zn^{2+}.

Figure S4. Thermogram for titration of AFP peptide with Zn^{2+}.

Figure S5. Stacked plot of the 1D ^{1}H NMR spectra of apo AFP-peptide recorded at various temperatures.

Figure S6. Comparison of H(α) shifts with random coil shifts for the apo AFP peptide, species A and species B.

Figure S7. Comparison of proton chemical shifts in Zn-bound species A and B with those obtained for the apo AFP-peptide.

Figure S8. Models for possible zinc-binding modes for the AFP-peptide.

Materials and Methods

1. Expression and purification of recombinant human AFP

Recombinant AFP proteins were expressed using the EasySelect™ *Pichia* Expression Kit (ThermoFisher Scientific). To enable this, the native AFP gene sequence was cloned into a *P. pastoris* expression vector, pPicZα (ThermoFisher Scientific) immediately downstream and in-frame with the α-factor secretion signal from *S. cerevisiae* to target the protein for extracellular expression. The plasmid encoding AFP was linearised using the *PmeI* restriction enzyme and transformed into electrocompetent X-33 *P. pastoris* cells (ThermoFisher Scientific). Transformed cells were grown under Zeocin selection and genomic DNA was isolated from individual Zeocin resistant clones. Successful integration of recombinant DNA into the P. pastoris genome was confirmed by PCR using primers which border the AFP gene. The 5' AOX1 sequencing primer was used as the forward primer and the 3'AOX1 sequencing primer was used as the reverse primer. The following thermocycling conditions were used; 1 cycle of 95°C, 30 sec; followed by 30 cycles of 95°C, 30 sec; 54°C, 1 min; 68°C, 2 min; and 1 cycle of 72°C, 5 min. *P. pastoris* transformants demonstrating successful expression of recombinant AFP were used to inoculate 10 mL BMGY (1% yeast extract, 2% peptone, 0.1 M potassium phosphate, pH 6.0, 1.34% yeast nitrogen base, 0.0004% biotin, 1% glycerol) media with 100 μg/mL of Zeocin. This starter culture was then transferred into 300 mL BMGY media with 100 μg/mL Zeocin. After 24 h, cells were separated from the growth media by centrifugation at 3000 rpm for 10 min at 25 °C. Pelleted cells were resuspended in 2 L of BMGY media divided between 4 × 2 L baffled flasks (each flask containing 500 mL). The flasks were incubated in a shaker incubator at 28°C with shaking at 200 rpm. Cultures were grown for five days. To each culture, pure sterilised methanol was added every 24 h to a final concentration of 0.5% methanol. After five days, cells were removed from the expression medium by centrifugation at 8000 rpm for 30 min at 4°C. The supernatant was vacuum filtered and concentrated using a polyethersulfone (PES) membrane filter with a molecular weight cut-off (MWCO) of 30 kDa (Sartorius, Epsom, UK). Finally, the medium was brought to pH 7.2 by adding 0.02 M sodium phosphate with 1 M NaCl, pH 7.0.

Recombinant AFP was purified using a HiTrap chelating HP 5 mL column (Cytiva, Little Chalfont, UK) run using an ÄKTA Pure FPLC system (Cytiva). The column was charged with $CuSO_4$ and pre-equilibrated with five column volumes (CVs) of binding buffer (0.02 M sodium phosphate, 1 M NaCl at pH 7.2). The supernatant from the expression medium was loaded onto the column and washed with 20 CVs of binding buffer. Recombinant AFP was eluted with a linear gradient of NH_4Cl; the elution buffer was 20 mM sodium phosphate, 1 M NH_4Cl at pH 7.2. The eluted fractions were analysed using Coomassie-stained SDS-PAGE to evaluate their purity. Fractions containing a band corresponding to AFP were pooled and concentrated using a PES membrane filter with MWCO of 30 kDa (Sartorius, Epsom, UK). The concentrated fractions were purified further by size exclusion chromatography using a HiLoad Superdex-75 column (Cytiva) attached to the ÄKTA Pure system. The column was equilibrated in 10 mM Tris, 150 mM NaCl at pH 7.4. Eluate fractions containing AFP were identified using SDS-PAGE, pooled and concentrated using a PES membrane centrifugal filter (MWCO=30 kDa; Satorius, Epsom, UK). The protein was >95% pure (SDS-PAGE), and full sequence coverage was observed by peptide mass fingerprinting. Protein levels were quantified using a bicinchoninic acid protein concentration assay kit (ThermoFisher Scientific).

2. Isothermal titration calorimetry

All reagents used were of analytical grade or better. ITC experiments were performed at 298.0 ± 0.1 K on MicroCal VP-ITC instruments (Malvern Panalytical, Malvern, UK). Peptide samples were prepared with the same buffer stock solution (50 mM Tris-Cl buffer with 50 mM NaCl, pH 7.2) used in the zinc titration ITC experiments. Human recombinant AFP was prepared and titrated in a slightly different buffer solution (50 mM Tris-Cl buffer with 140 mM NaCl, pH 7.4). All samples were degassed by being stirred under vacuum before use. The solutions in the titration cells were stirred at 300 rpm by the syringe to ensure rapid mixing. In the ITC experiments for the peptide (25 μM), 30 injections of 6 or 8 μL of a 333 μM zinc solution ($ZnCl_2$, Sigma) were made into the cell containing the sample solution. For the protein (20 μM), 32

injections of 5 μL of a 300 μM zinc solution were made. In each case, a background titration, consisting of the identical titrant solution but only the buffer solution in the sample cell, was subtracted from each experimental titration to account for heat of dilution. Data were analysed with Affinimeter software (Santiago de Compostela, Spain), and the fitting curves were calculated according to binding site models provided with the software.

3. Inductively coupled plasma-optical emission spectroscopy (ICP-OES)

An AFP-peptide sample was prepared in ammonium acetate (NH_4OOCCH_3; 10 mM, pH 7.4). A ca. 10-fold excess of $ZnCl_2$ was added to each sample, followed by incubation for 2 h at 310 K. To remove unbound and weakly bound metal ions, the sample was passed through a desalting column (G25-Sephadex, PD10, Cytiva). Samples were appropriately diluted with 0.1 M ultrapure HNO_3 (prepared in-house by sub-boiling point distillation). Zn and S contents were simultaneously determined on a Perkin Elmer Optima 3000DV ICP-OES spectrometer, which had been calibrated with standards of concentrations between 0 to 2.5 ppm for Zn and 0 to 5 ppm for S, prepared gravimetrically from high grade commercial stocks (1000 ppm, Fluka). The data, given in units of mg/L (ppm), were divided by the respective atomic masses, which gives the concentration in mmol of the given element in the sample. The AFP-peptide and full-length AFP concentration was obtained by dividing the mmol of sulfur by the numbers of sulfurs in the peptide (=2). Subsequently the metal to peptide ratio was calculated.

4. Electrospray ionisation mass spectrometry

Samples of 15-25 μM peptide were prepared in 10 mM NH_4OOCCH_3 buffer with 10% (v/v) methanol (pH 7.4). A sample of zinc-loaded AFP-peptide was prepared by adding a 10-fold excess of zinc to AFP-peptide sample, followed by incubation for 2 h at 310 K in 10 mM NH_4OOCCH_3 buffer with 10% (v/v) methanol (pH 7.4). Mass spectra were recorded on a MicrOTOF instrument (Bruker Daltonics) with electrospray ionisation (ESI) source. The

samples were directly infused into the source using a syringe pump with a flow rate of 240 μL h^{-1}. Mass spectral data were acquired for 2 min in the positive mode over the m/z range of 0-3000, using the following settings: Source temperature: 473 K; voltages: capillary exit: 150 V, end plate: 4000 V, skimmer 1: 50 V, skimmer 2: 23.5 V, hexapole 1: 24 V, hexapole 2: 20.9 V. Mass spectra were processed using Compass Data Analysis software (v.4.4, Bruker Daltonics). Theoretical mass spectra were calculated using the EnviPat server at https://www.envipat.eawag.ch/ and plotted in Microsoft Excel.

5. 1D and 2D ^{1}H NMR experiments

NMR samples (1.0 mM) were prepared in 50 mM [D_{11}]Tris-Cl (99% D; CK Gas, UK), 50 mM NaCl, 10% D_2O (99%, Sigma-Aldrich, UK) at pH 7.1. Additions of Zn^{2+} were made as small aliquots of 50 mM stock solutions of $ZnCl_2$. The pH was adjusted with 1 M HCl or NaOH. The pH values of the samples were monitored before and after Zn^{2+} additions.

1D ^{1}H NMR spectra were recorded at 278 K using a Bruker DRX700 spectrometer equipped with a TCI cryoprobe operating at 700.13 MHz for ^{1}H. The H_2O peak at 4.528 ppm was suppressed using excitation sculpting with pulsed-field gradients.[1] Spectra were collected using 4k complex data points over 128 scans. FIDs were transformed with 16k data points and further processed using exponential multiplication. Spectra were processed using TOPSPIN 2.1 (Bruker, UK).

2D datasets were acquired on at 278 K on the same instrument. 2D [^{1}H,^{1}H] nuclear Overhauser enhancement (NOESY; 500 ms mixing time) and total correlation (TOCSY; mmixing time 64 ms) spectra were acquired with 32 scans with 4k data points over a spectral width of 13 ppm in the F_2 dimension and 512 increments in the F1 dimension. Raw data were apodised using squared sine-bell functions and Fourier-transformed with 2k $\times$ 2k data points. Two-dimensional data were processed with TOPSPIN 2.1 (Bruker, UK). Spectra were

calibrated using the residual H_2O peak.[2] The analysis of all 2D NMR data was carried out using SPARKY v.3.114 software.[3]

6. Molecular modelling

Starting models of zinc-bound AFP-peptide were initially generated using the AlphaFold server (https://alphafoldserver.com/) by submitting the peptide sequence and selecting a Zn^{2+} ion to form a complex. The 5 models returned were inspected and further refined in Chimera v. 1.11[4] as follows. The Cys251-Cys259 sulfurs were connected by a bond. Tetrahedral coordination spheres were completed by adding an oxygen atom, using the "Metal Geometry" sub-routine in Chimera. Next, hydrogen atoms and charges (Gasteiger) were added using the AnteChamber[5] routines incorporated in Chimera. Tautomers of histidine residues were specified manually. The resulting models were then refined by energy minimisation (100 steps of steepest descent and 10 steps conjugate gradient) using a customised Amber14 forcefield. We noted that some of the AlphaFold models featured the sulfur of one of the cystines as coordinating, even though in each case, the two sulfurs were in bonding distance to each other (oxidised sulfurs have negligible zinc-binding ability). The subsequent energy minimisation typically corrected this mistake.

The final refined model for full-length AFP was also generated in Chimera, starting from the published pdb entry 8x1n. As for the peptides, an oxygen atom was added to the zinc coordination sphere, followed by addition of hydrogen atoms and charges. A first round of energy minimisation involved the zinc ion and surrounding residues (ligands plus Asp258) only, followed by a second round involving the entire protein.

Table S1. ITC data for full-length AFP, as obtained using Affinimeter software. The final model is highlighted in blue. The last column gives goodness-of-fit values, with higher numbers indicating better fits.

Model	n(1 or 2)	Error(n)	K_a [M^{-1}]	Error (K)	ΔH [$kJ.mol^{-1}$]	Error (ΔH)	GoF
1 Set of sites	1.16	0.01	$2.315x10^5$	$8.88x10^3$	-11560	150	5%
2 Sets of sites *)	0.915	0.005	$1.776x10^7$	$2.77x10^6$	-8596	8.9	56%
	0.05	0	$2.449x10^4$	$3.10x10^2$	-194780	680	
2 Sets of sites (n_2 fixed to 1)	0.93	0.01	$2.363x10^7$	$1.40x10^6$	-8528	11.1	59%
	1	--	$5.668x10^4$	$1.1x10^3$	-6776	41.5	
1 independent site	--	--	$1.960x10^5$	$4.14x10^3$	-12369	5.4	5%
2 independent sites	--	--	$1.922x10^7$	$1.20x10^6$	-8635	9.4	46%
	--	--	$4.367x10^4$	$4.25x10^3$	-8375	60.1	

*) This model did not converge and gave unreasonable values for ΔH and n2 (highlighted in red; lower bound for n2 was reached without convergence).

Table S2. Observed and theoretical masses for apo and zinc-bound AFP peptide (20 μM, 10 mM NH_4OOCCH_3, pH 7.4, 10% methanol).

	Neutral (Da)				+3 charge state (m/z)			
	Mono-isotopic		Most abundant		Mono-isotopic		Most abundant	
	Obs.	Theo.	Obs.	Theo.	Obs.	Theo.	Obs.	Theo.
Apo	2128.99	2128.92	2129.99	2129.91	710.69	710.65	711.02	710.98
Zn_1	2191.20	2190.83	2193.10	2192.83	731.41	731.28	732.06	731.95
Zn_2	2253.11	2252.74	2257.07	2256.71	752.04	751.92	753.37	753.26

Table S3. ITC data for AFP peptide as obtained using Affinimeter software. The final model is highlighted in blue. The last column gives goodness-of-fit values, with higher numbers indicating better fits.

Model	n	Error(n)	K_a [M^{-1}]	Error (K)	ΔH [$kJ.mol^{-1}$]	Error (ΔH)	GoF
1 Set of sites	1.033	0.001	$4.446x10^5$	$2.92x10^3$	-4646	5.9	75%
2 Sets of sites *)	0.975	0.005	$6.483x10^5$	$3.41x10^4$	-4428	6.1	90%
	0.05	0.05	$2.926x10^4$	$3.26x10^3$	-15655	2217	
2 sets of sites (n_2=1)	0.97	0.01	$1.258x10^6$	$7.28x10^4$	-4456	3.8	91%
	1-fixed	--	$4.287x10^4$	$4.23x10^3$	-690	7.9	
1 independent site	--	--	$4.446x10^5$	$2.92x10^3$	-4646	5.9	75%
2 independent sites	--	--	$1.278x10^6$	$7.01x10^4$	-4330	7.7	90%
	--	--	$4.115x10^4$	$6.9x10^3$	-842	10.3	

*) This model did not converge and gave unreasonable values for ΔH and n2 (highlighted in red; lower bound for n2 was reached without convergence).

Table S4. ^{1}H NMR chemical shifts for apo peptide (700.13 MHz,1 mM peptide in 50 mM [D_{11}]Tris-Cl, 50 mm NaCl, pH 7.1, 278 K)

**Residue**	**NH**	**H(α)**	**H(β)**	**H(γ)**	**H(δ)**	**H(ε)**
Ala245	7.955	3.736	0.842	-	-	-
His246	7.998	4.136	2.570	-	6.438	7.374
Val247	7.491	3.515	1.484	0.338	-	-
His248	7.976	4.139	2.556; 2.623	-	6.531	7.351
Glu249	8.040	3.751	1.516; 1.450	1.699	-	-
His250	8.199	4.165	2.669	-	6.556	7.385
Cys251	8.004	4.237	2.753; 2.581	-	-	-
Ser252	8.352	3.968	3.471	-	-	-
Arg253	7.752	3.898	1.506; 1.339	1.199	2.746	n.d.
Gly254	7.875	3.561; 3.442	-	-	-	-
Asp255	7.862	4.208	2.305; 2.221	-	-	-
Val256	7.578	3.678	1.697	0.464	-	-
Leu257	7.930	3.809	1.236	1.096	0.463	0.404
Asp258	7.831	4.111	2.241; 2.199	-	-	-
Cys259	7.702	4.159	2.769; 2.620	-	-	-
Leu260	7.919	3.885	1.233	1.097	0.450	0.394
Gln261	7.996	3.838	1.643; 1.524	1.896	-	7.178; 6.475
Asp262	8.070	4.107		-	-	-
Gly263	8.047	3.451; 3.402	-	-	-	-

Table S5. ^{1}H NMR chemical shifts for Zn-bound species A (700.13 MHz,1 mM peptide and 1 mM Zn^{2+} in 50 mM [D_{11}]Tris-Cl, 50 mm NaCl, pH 7.1, 278 K)

Residue	NH	H(α)	H(β)	H(γ)	H(δ)	H(ε)
Ala245	7.891	3.568	0.712	-	-	-
His246	8.237	3.964	3.184; 2.368	-	6.735	7.469
Val247	7.051	3.437	1.683	0.529	-	-
His248	7.955	4.553	2.402; 2.905	-	6.167	7.688
Glu249	8.421	4.696	1.460; 1.391	2.072	-	-
His250	8.386	4.057	2.163;2.072	-	6.078	7.159
Cys251	9.750	3.660	2.783; 2.429	-	-	-
Ser252	7.372	4.038	3.597; 3.488	-	-	-
Arg253	n.d.	3.579	1.389; 1.342	1.247; 1.184	2.767	n.d.
Gly254	n.d.	3.591; 3.477	-	-	-	-
Asp255	7.363	4.476	2.763; 2.529	-	-	-
Val256	7.854	3.984	1.966	0.433; 0.459	-	-
Leu257	7.667	3.757	1.180	1.075	0.331	0.241
Asp258	6.885	4.122	2.554; 2.063	-	-	-
Cys259	7.218	4.387	3.136; 1.825	-	-	-
Leu260	9.150	3.871	1.252	1.111	0.493	0.342
Gln261	7.712	3.865	1.619; 1.465	1.851	-	7.046;6.353
Asp262	8.194	4.095	2.244	-	-	-
Gly263	8.045	3.392; 3.470	-	-	-	-

Table S6. ^{1}H NMR chemical shifts for Zn-bound species B (700.13 MHz,1 mM peptide and 1 mM Zn^{2+} in 50 mM [D_{11}]Tris-Cl, 50 mm NaCl, pH 7.1, 278 K).

Residue	NH	H(α)	H(β)	H(γ)	H(δ)	H(ε)
Ala245	7.938	3.807	0.907	-	-	-
His246	8.428	4.141	2.560; 2.128	-	6.698	7.422
Val247	8.646	3.586	1.742	0.462	-	-
His248	7.642	4.606	2.221; 2.728	-	6.078	7.158
Glu249	6.583	3.981	1.434	1.733	-	-
His250	8.619	3.820	2.031; 2.436	-	6.666	7.440
Cys251	10.340	3.605	2.816; 2.434	-	-	-
Ser252	6.984	4.062	3.712; 3.506	-	-	-
Arg253	n.d.					
Gly254	n.d.					
Asp255	n.d.					
Val256	7.640	3.799	1.910	0.452	-	-
Leu257	7.941	3.806	1.359	1.003	0.328	0.240
Asp258	6.683	4.444	2.772; 1.986	-	-	-
Cys259	6.795	4.380	3.426;1.760		-	-
Leu260	8.097	3.840	1.198	1.125	0.423	-
Gln261	8.138	3.900	1.646; 1.511	1.902	-	7.043; 6.450
Asp262	8.081	4.103	2.245	-	-	-
Gly263	8.069	3.464; 3.407	-	-	-	-

Table S7. Angles and distances for zinc-binding site in full-length AFP after force-field-driven optimisation of geometry through energy minimisation.

Angle (degrees)		Expected for TBP[a)]	Distances (Å)	
H4(Ne1)-Zn-H246(Ne1)	84.30	90	H4-Zn	2.10
H4(Ne1)-Zn-H250(Ne1)	155.56	180	H246-Zn	2.11
H4(Ne1)-Zn-D262(Od2)	97.77	90	H250-Zn	2.06
H246(Ne1)-Zn-H250(Ne1)	83.10	90	D262-Zn	1.82
H246(Ne1)-Zn-D262(Od2)	108.92	120	water(O)-Zn	1.93
H250(Ne1)-Zn-D262(Od2)	106.10	90		
H4(Ne1)-Zn-water(O)	86.02	90		
H246(Ne1)-Zn-water(O)	137.80	120		
H250(Ne1)-Zn-water(O)	89.36	90		
D262(Od2)-water(O)	113.07	120		

a) TBP: trigonal-bipyramidal

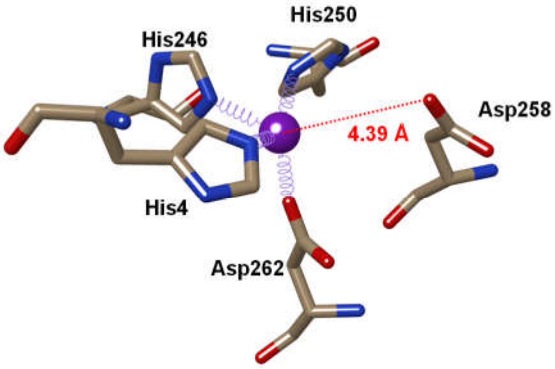

Angle (degrees)		Expected for 4-coordinate (degrees)
H4(Nε1)-Zn-H246(Nε1)	93.49	109.47
H4(Nε1)-Zn-H250(Nε1)	133.73	
H4(Nε1)-Zn-D262(Oδ2)	114.71	
H246(Nε1)-Zn-H250(Nε1)	69.86	
H246(Nε1)-Zn-D262(Oδ2)	107.08	
H250(Nε1)-Zn-D262(Oδ2)	111.45	

Distances (Å)		Expected (Å)
H4(Nε1)-Zn	2.41	2.00
H246(Nε1)-Zn	2.35	
H250(Nε1)-Zn	2.30	
D262(Oδ2)-Zn	2.45	2.04

Figure S1. The geometry (angles and distances) of the modelled Zn^{2+} site in pdb 8X1N. Expected distances are taken from reference 6. Angles are closer to those expected for trigonal-bipyramidal geometry (angles of 120° and 90°), with the fifth ligand missing. It may be envisaged that the coordination sphere may be completed by a water molecule. Whilst a simple rotation around the Cα-Cβ bond would bring an oxygen of Asp258 much closer (2.68 Å), the resulting model is not consistent with the published electron density map (not shown).

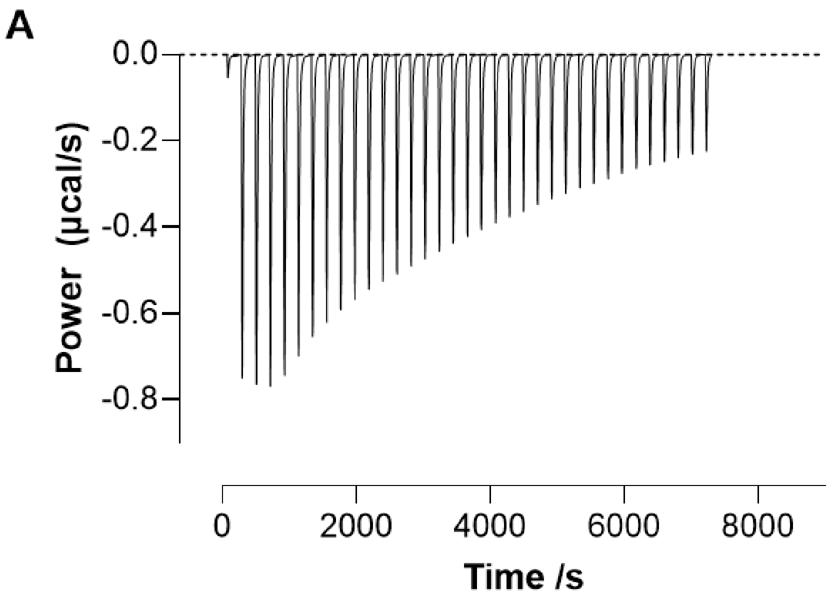

Figure S2. Thermogram for titration of full-length AFP (20 μM) with Zn^{2+} (50 mM Tris-Cl, 140 mM NaCl, pH 7.4, 298 K).

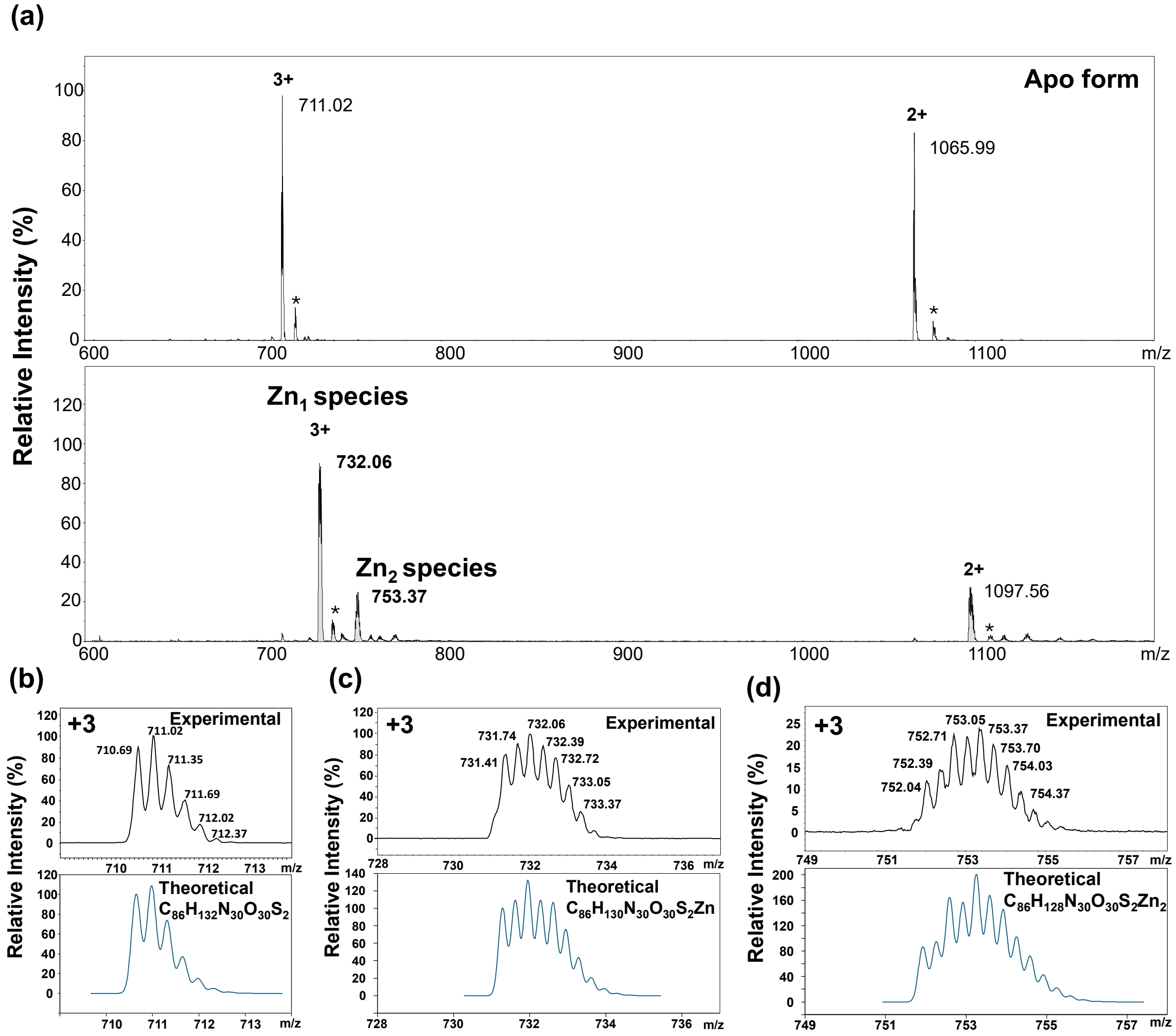

Figure S3. ESI-MS analysis of AFP peptide in its apo form and after incubation with Zn^{2+}. (a) Full scans of AFP peptide (top) and after incubation with $ZnCl_2$ (bottom)(20 μM peptide, 10 mM NH_4OOCCH_3, pH 7.4, 10% methanol). The peaks marked with an asterisk are Na^+ adducts. (b) – (d) Experimental and theoretical MS data for the +3 charge states for apo (b), Zn_1 (c), and Zn_2 (d) species. Models were generated using the EnviPat server at https://www.envipat.eawag.ch, using a resolution of 3000 FWHM (full width at maximum height), and data were plotted in MS Excel.

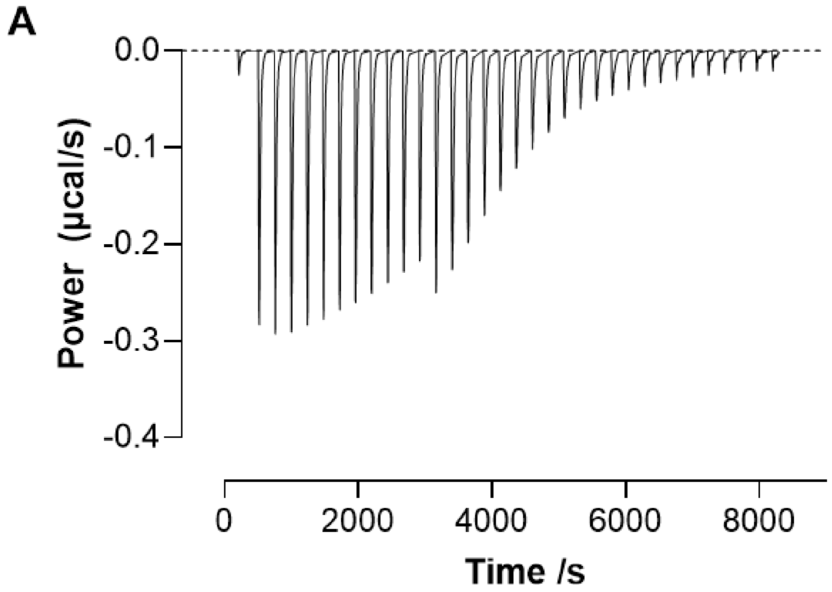

Figure S4. Thermogram for titration of AFP peptide (25 μM) with Zn^{2+} (50 mM Tris-Cl, 50 mM NaCl, pH 7.2, 298 K).

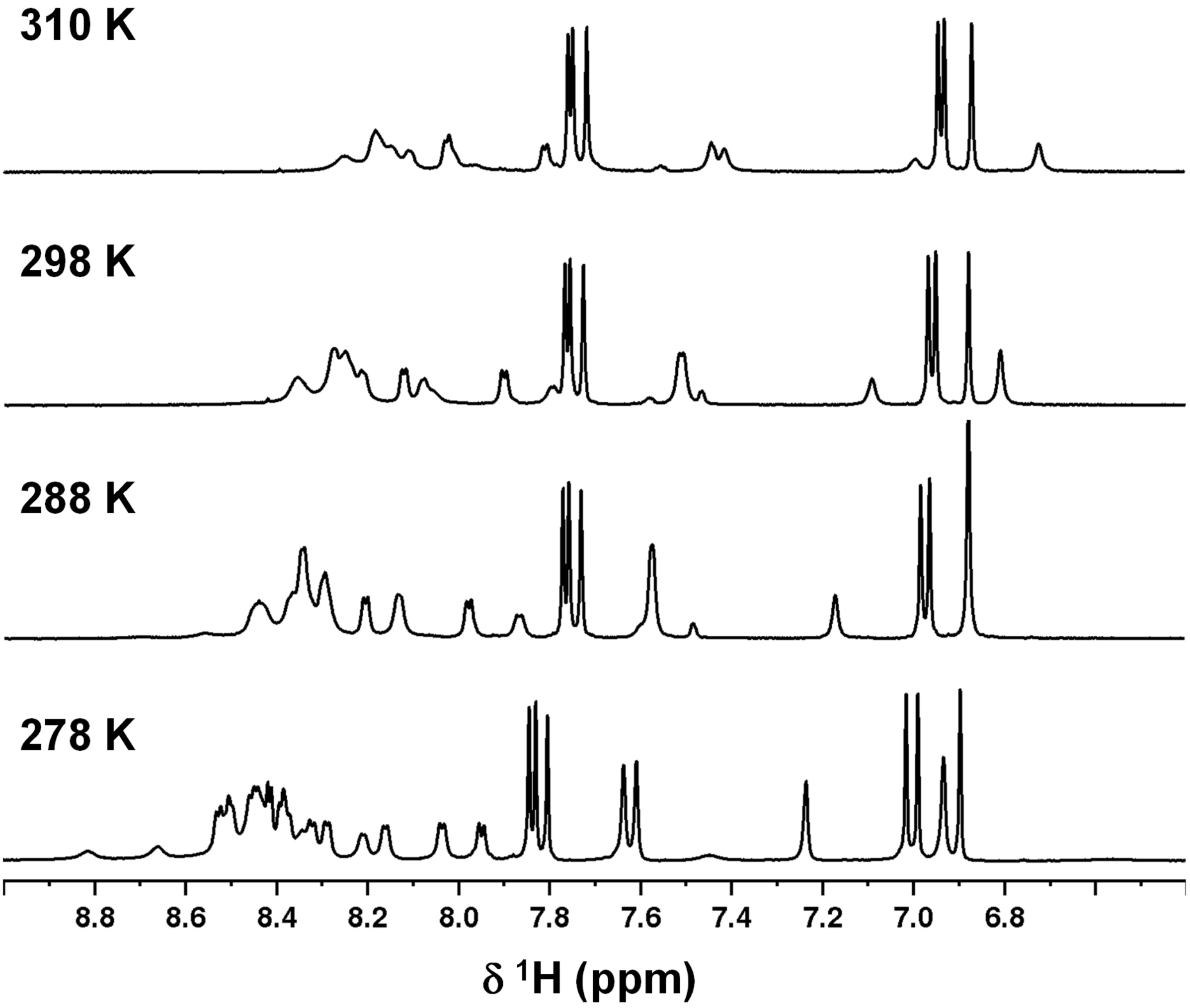

Figure S5. Stacked plot of the 1D ^{1}H NMR spectra of apo AFP-peptide recorded at various temperatures. Spectra were recorded on a Bruker DRX 700 MHz spectrometer (1 mM peptide, 50 mM [D_{11}] Tris, 50 mM NaCl (pH 7.1) and 10% D_2O).

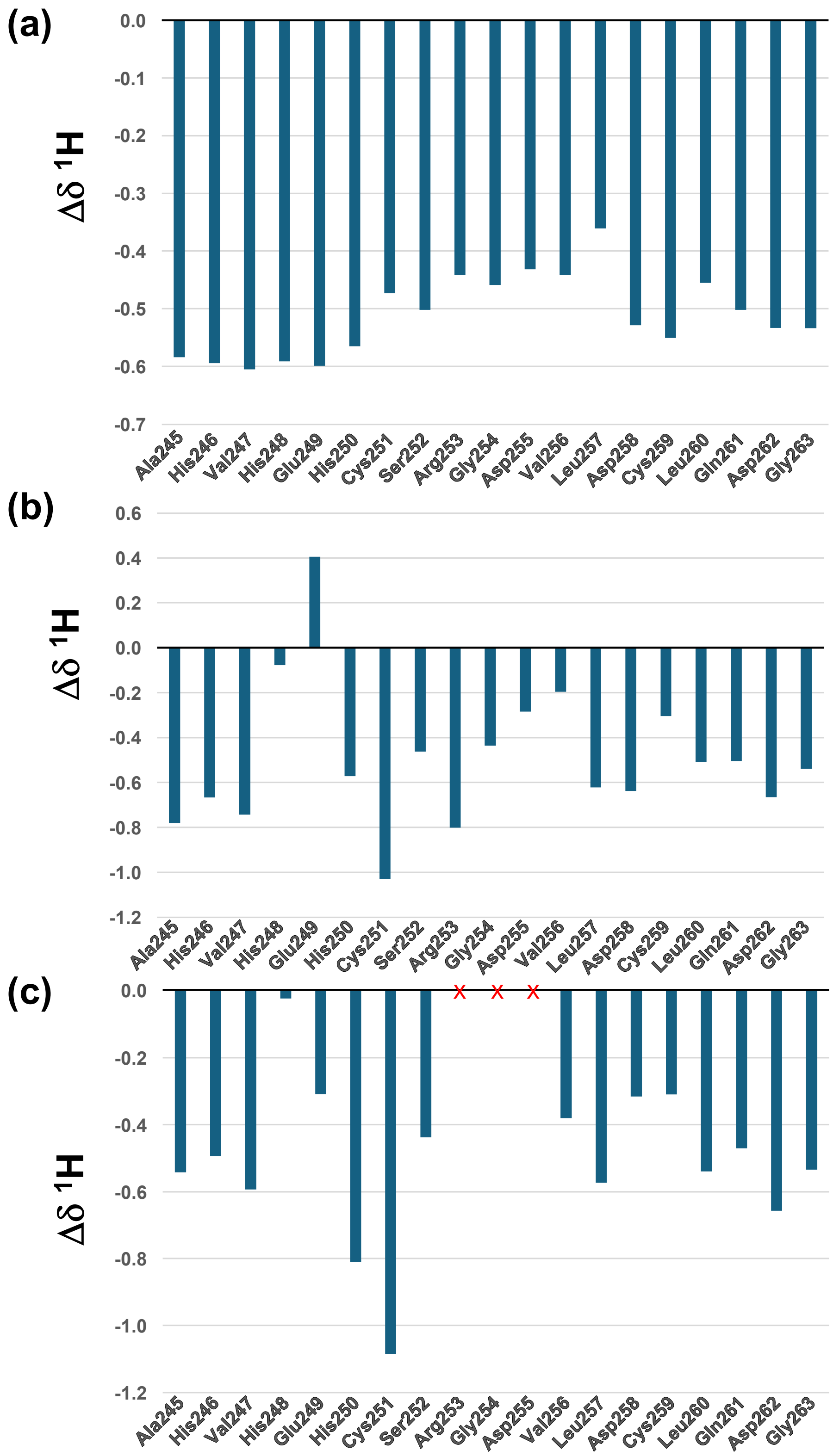

Figure S6. Comparison of H(α) shifts with random coil shifts for (a) the apo AFP peptide, (b) species A and (c) species B ($\Delta\delta$ ^{1}H = δ(experimental) – δ(random coil)). The predominantly negative values of $\Delta\delta$ ^{1}H are consistent with α-helical character. Red crosses indicate missing data for unassigned residues in species B. Random coil data were taken from reference 7.

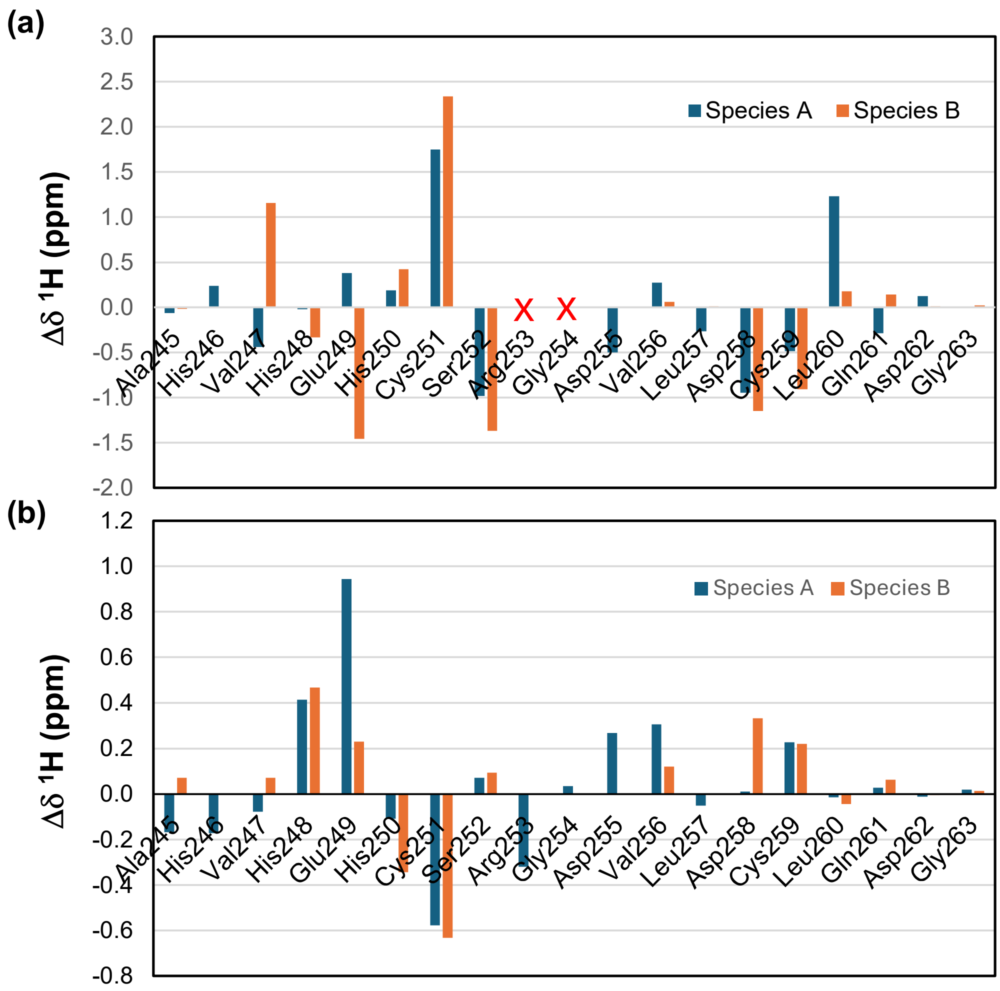

Figure S7. Comparison of proton chemical shifts in Zn-bound species A and B with those obtained for the apo AFP-peptide. (a) NH protons, and (b) H(α) protons. Red crosses indicate absence of chemical shift data for species A and B.

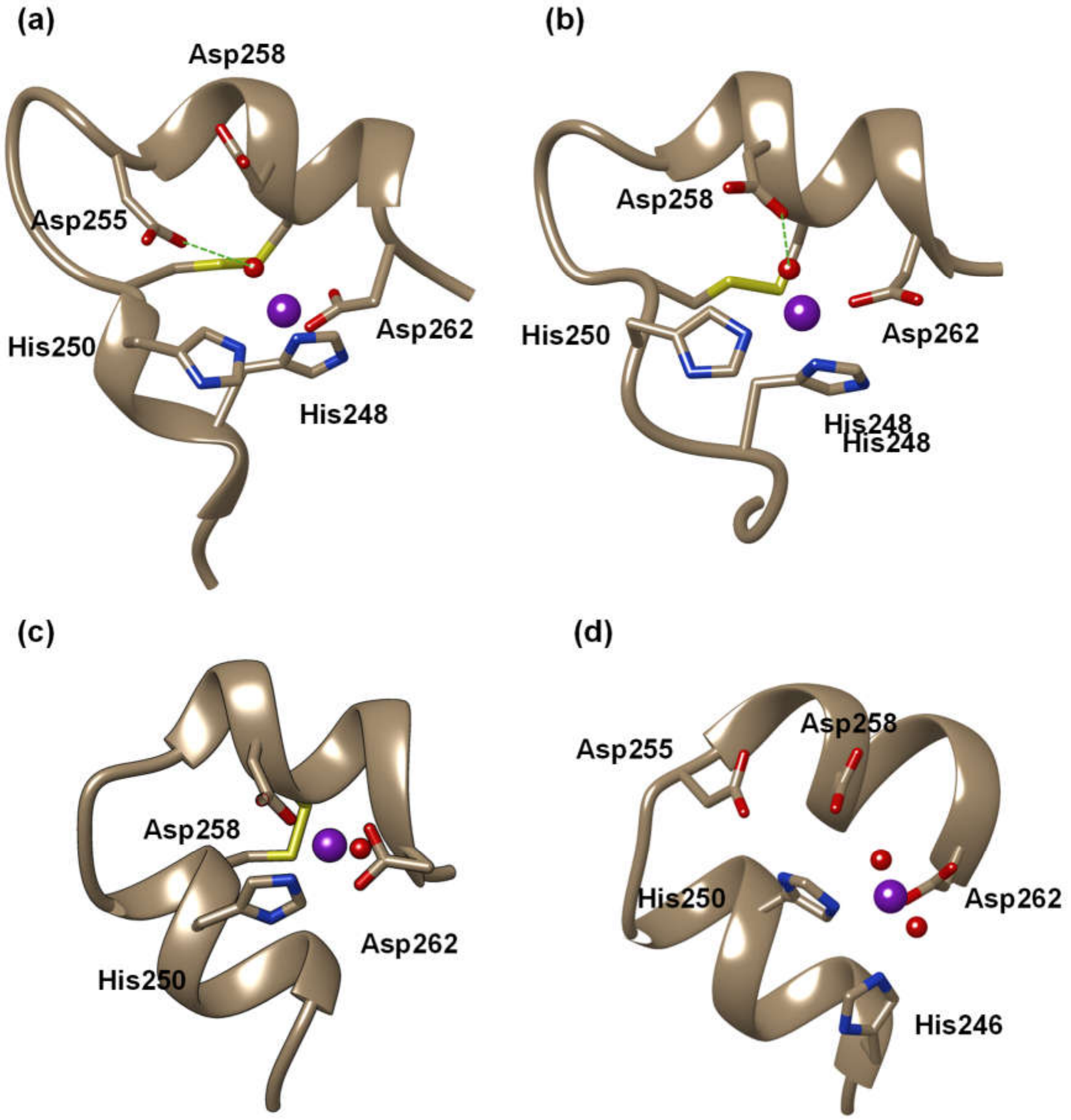

Figure S8. Models for possible zinc-binding modes for the AFP-peptide. Starting models (a) to (c) for AFP-peptide-Zn complexes were generated by AlphaFold and, after adding a water molecule to complete tetrahedral coordination, subjected to further energy minimisation in Chimera v. 1.11, using a customised AMBER forcefield (based on ff14SB). Models (a) and (b) feature the same ligand set in the first coordination shell, but this differs from that realised in the cryo-EM structure, which has His246 instead of His248. Accommodation of His248 coordination requires unwinding of the first α-helix, which is unlikely to happen in the full-length protein. Both models feature an H-bond from the water molecule to either Asp255 or Asp258. Model (c) features intact α-helices, but neither His246 nor His248 are bound. Instead, Asp258 features as the third protein-derived ligand. The bound water is not hydrogen bonded to an amino acid sidechain. Model (d) was generated from the cryo-EM structure and features the three ligands bound in that structure; two water molecules were added after attempts to refine models with one water molecule failed. We also attempted to generate a model of the peptide based on the cryo-EM structure, with a binding site composed of His246, His250 and Asp262. Invariably, attempts to optimise these models (with either 1 or 2 bound water molecules added) led to unrealistic geometries.

References

1. T. L. Hwang and A. J. Shaka, *J. Magn. Reson. Series A*, 1995, **112**, 275-279.
2. D. S. Wishart, C. G. Bigam, J. Yao, F. Abildgaard, H. J. Dyson, E. Oldfield, J. L. Markley and B. D. Sykes, *J. Biomol. NMR*, 1995, **6**, 135-140.
3. T. D. Goddard and D. G. Kneller, SPARKY 3, University of California, San Francisco.
4. E. F. Pettersen, T. D. Goddard, C. C. Huang, G. S. Couch, D. M. Greenblatt, E. C. Meng, T. E. Ferrin, *J. Comput. Chem.,* 2004, **25**, 1605-1612.
5. J. Wang, W. Wang, P. A. Kollman and D. A. Case, *J. Mol. Graph. Model.* **25**, 247-260.
6. M. M. Harding, *Acta Crystallogr. D Biol. Crystallogr.*, 2001, **57**, 401-411
7. D. S. Wishart, *Progress in Nuclear Magnetic Resonance Spectroscopy*, 2011, **58**, 62-87.
